# Regional Aggressive Root Resorption Caused by Neuronal Virus Infection

**DOI:** 10.1155/2012/693240

**Published:** 2012-10-14

**Authors:** Inger Kjær, Carsten Strøm, Nils Worsaae

**Affiliations:** ^1^Department of Orthodontics, Institute of Odontology, Faculty of Health Sciences, University of Copenhagen, 20 Norre Alle, 2200 Copenhagen, Denmark; ^2^Private Dental Practice, Lyngbyvej 133, 2100 Copenhagen, Denmark; ^3^Department of Oral and Maxillofacial Surgery, Copenhagen University Hospital, Rigshospitalet, 2100 Copenhagen, Denmark

## Abstract

During orthodontic treatment, root resorption can occur unexplainably. No clear distinction has been made between resorption located within specific regions and resorption occurring generally in the dentition. The purpose is to present cases with idiopathic (of unknown origin) root resorption occurring regionally. Two cases of female patients, 26 and 28 years old, referred with aggressive root resorption were investigated clinically and radiographically. Anamnestic information revealed severe virus diseases during childhood, meningitis in one case and whooping cough in the other. One of the patients was treated with dental implants. Virus spreading along nerve paths is a possible explanation for the unexpected resorptions. In both cases, the resorptions began cervically. The extent of the resorption processes in the dentition followed the virus infected nerve paths and the resorption process stopped when reaching regions that were innervated differently and not infected by virus. In one case, histological examination revealed multinuclear dentinoclasts. The pattern of resorption in the two cases indicates that innervation is a factor, which under normal conditions may protect the root surface against resorption. Therefore, the normal nerve pattern is important for diagnostics and for predicting the course of severe unexpected root resorption.

## 1. Introduction

Deviations in the dentition can occur regionally (affecting specific teeth) or generally (affecting several teeth in the dentition).

**Figure 1:**
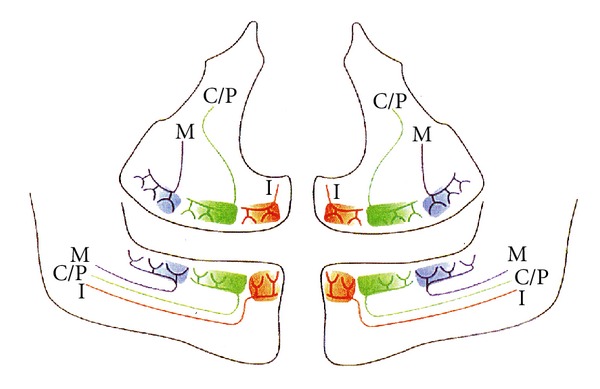
Schematic illustration of a panoramic radiograph with peripheral nerves innervating the different tooth groups. Red colour (I) illustrates the innervation of maxillary and mandibular incisors. Green colour (C/P) illustrates the innervation of maxillary and mandibular canines and premolars. Blue colour (M) illustrates the innervation of maxillary and mandibular molars. This illustration is reproduced with permission from Acta Odontologica Scandinavica [7].

An interesting case was published in 1995 [1] demonstrating a right-sided maxillary underdevelopment of teeth and jaw combined with right-sided hearing loss provoked by mumps virus. This observation motivated the search for clarity regarding jaw innervation. One of the results was an innervation diagram, which was first published in 1998 [2] (Figure 1). In particular, the innervation of the mandible with innervation paths to the different tooth groups, published in 1996 [3], has made it possible to understand that the innervation to the incisors and to the canines/premolars and also to the molars is caused by different nerve paths in the maxilla and mandible. The innervation diagram has made it possible to understand how innervation is involved in eruption [4] showing a close connection between eruption times within the three different tooth groups in each jaw, but not between the tooth groups.

The regional pattern observed in retention of permanent teeth has also been explained from the innervation pattern when regionally affected by virus infection [5]. Furthermore, segmental odontomaxillary dysplasia seems to occur in regions correlated to the innervation patterns [6]. Interrelationship between the central nervous system and the surrounding bone and the relationship between the peripheral nervous system and jaw bone and teeth have been designated neuro-osteology [7]. A relationship between prenatal peripheral nervous tissue and the initial osseous bone formation in the craniofacial skeleton was first described in 1990 [8]. In 1992, Lerner described the interrelationship between neuropeptides and bone metabolism at a molecular level [9]. Thus, the term neuro-osteology is verified in histological as well as molecular studies.

**Figure 2:**
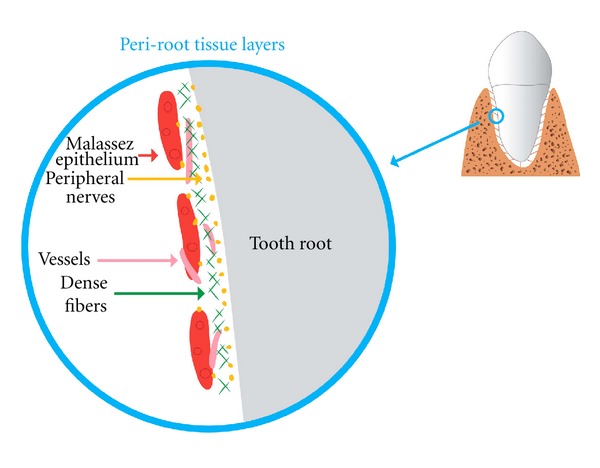
The figure in the circle is an enlargement of the area shown on the tooth in the upper right corner. The cell layers close to the root's periodontium, also known as the periroot sheet layers, are demonstrated in the circle. Nerve tissue: yellow. Tense fibre layer: green. Malassez epithelial cells: dark red. Vessels: light red. This illustration is reproduced with permission from Orthodontic Waves [27].

In histological studies of the dental development, it has been shown that the initial dental development may begin without innervation [10], while the innervation is important at the time when the hard dental tissue is formed [11–13]. Recent studies have analyzed tissue components close to the root surface [14–16]. These studies have revealed that an innervation layer exists in close proximity to the root, covered by a layer of tight fibers and cell groups from the Malassez epithelium. It has also been demonstrated that the neuroendocrine cells are located in relation to the Malassez epithelium [17]. Similarly, a study by Yamashiro et al. [18] suggesting that sensory nerve innervation may have a regulatory role in maintenance of the epithelial rests of Malassez [18]. It has been a repeated question if one or all of these layers may offer protection against root resorption. The layers are schematically illustrated in Figure 2.

In Paget's disease, also named osteitis deformans, root resorptions have been observed [19, 20] and a viral aetiology has been discussed in a series of studies [21–25]. The characteristics of Paget's disease are the presence of focal areas of increased bone turnover containing enlarged hyperactive osteoclasts. Also a genetic predisposition has been in focus [26]. 

In orthodontic literature, a clear distinction has not been made between resorption located within specific regions and resorption occurring generally in the dentition. The distinction between resorption with a known cause and idiopathic root resorption (of unknown cause or spontaneous origin) has not been made. A main problem in orthodontics is that root resorption can occur unexplainably and it is, therefore, important to focus on causes rather than orthodontic forces that may provoke root resorption.

In the present paper, two cases of regional aggressive resorption are described, both seemingly with a viral aetiology.

## 2. Patient Cases

The paper describes diagnostics and treatment course for two patients referred to the Department of Orthodontics for elucidation and treatment plan.

### 2.1. Patient 1

**Figure 3:**
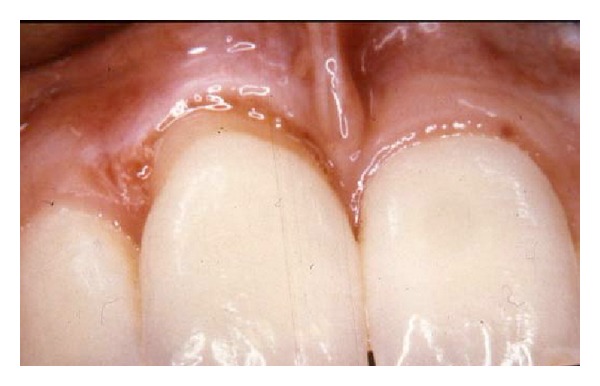
Patient 1: gingival retraction at the right maxillary central incisor after acute gingivitis in 1992.

**Figure 4:**
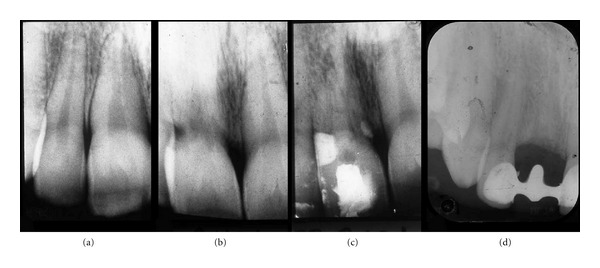
Patient 1: radiographs of the right maxillary central incisor region. (a) 1994. Seemingly normal periodontal conditions in the central incisor region. (b) 1997. Severe cervical resorptions have appeared in the central incisor. (c) August 1999. Severe resorption of the root of the maxillary central incisor. The condition resulted in extraction of the central incisor. (d) December 1999. The maxillary central incisor has been extracted and the alveolar bone level is normal.

The patient, female born in 1974, is referred in April 2000 for elucidation of a previous severe idiopathic resorption of the right maxillary permanent central incisor that had recently been extracted. For the consultation, the patient brought anamnestic information, a photograph from 1992 (Figure 3) and radiographs from 1994 (Figure 4(a)), 1997 (Figure 4(b)), and 1999 (Figures 4(c) and 4(d)) of the central incisor. The patient had first contacted the dentist in 1992 due to retraction of the gingival level as seen in Figure 3. Examination of the four radiographs revealed severe root resorption starting at the cervical level of the right central incisor gradually progressing to the whole root. In Figure 4(d), the normal alveolar bone level after extraction is seen.

**Figure 5:**
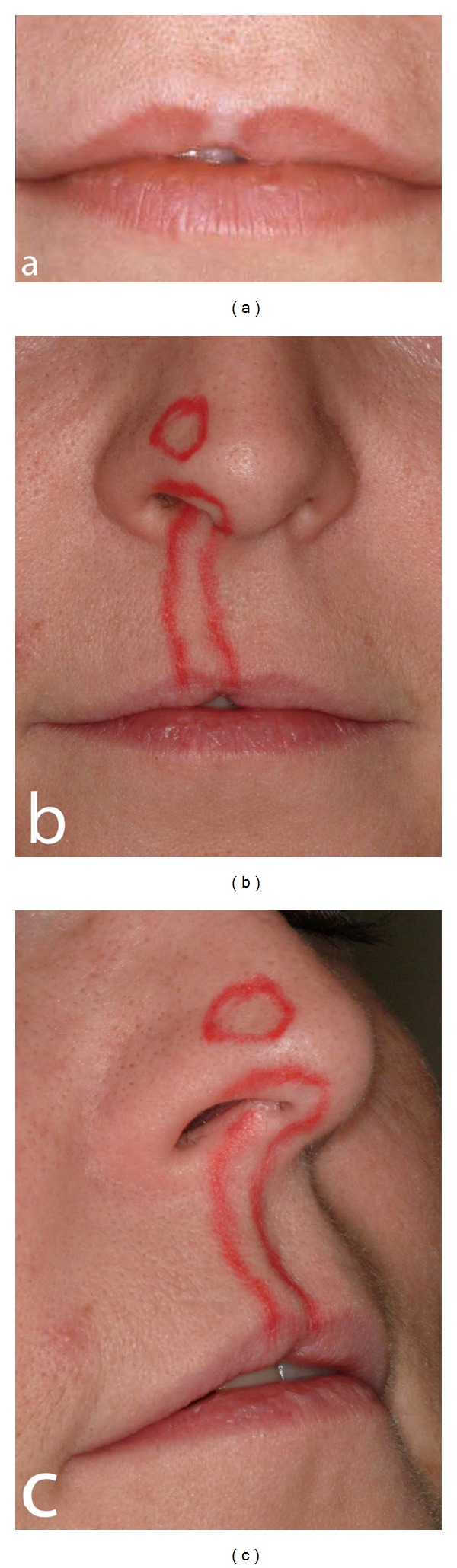
Patient 1: extraoral photographs. (a) The photograph shows retraction of the lip in the area of the central incisor. A white fibrous area is seen in the otherwise red prolabium in the same area. (b) and (c) Red colour marks the area of the skin at the upper lip and nose where the sensitivity is different (feeling of dead skin).

The extraoral examination in 2000 showed a pale scar tissue-like retraction of the upper lip just to the right of the midline in an otherwise normal red prolabium (Figure 5(a)). Furthermore, impaired sensitivity of the skin was observed in an area extending from the right prolabium to the right medial and lateral crus of the external nose. This affected skin area (marked by red colour) is demonstrated in Figures 5(b) and 5(c). Anamnestic information revealed that the patient had meningitis between the age of 8 and 9 and that the change in skin sensitivity began at the same time. 

**Figure 6:**
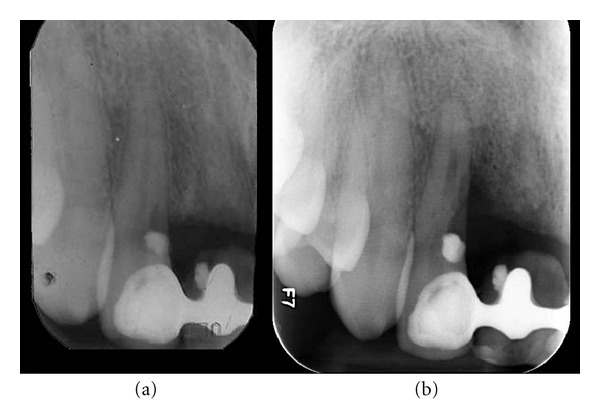
Patient 1: radiographs of the right maxillary central incisor region. (a) 2000. The radiograph demonstrates a filling in the mesial aspect of the lateral incisor and resorption below this filling. The alveolar bone level has not changed remarkably since the situation demonstrated in Figure 4(d). (b) 2012. The radiograph demonstrates severe bone loss of the alveolar bone mesially to the lateral incisor root. The resorption process below the filling has progressed.

A new radiograph of the incisor region obtained at the consultation in 2000 demonstrated an initial cervical root resorption of the right permanent lateral incisor (Figure 6(a)). The patient has then been followed annually since 2000 and radiographs of the incisor region have revealed a gradual progressive resorption of the root of the lateral incisor as well as of the alveolar bone facing the medial aspects of the root (demonstrated in the examination in 2012, Figure 6(b)). No tumor-like conditions like for instance giant cell granuloma were observed. 

#### 2.1.1. Conclusion

It is suggested that the meningitis virus has spread along the right nasopalatine nerve innervating the skin region and the right incisors and that this may be the explanation for the regional root resorption and bone destruction. If this is the explanation, it can be predicted that the destructive process is limited to the right incisor region. For visualization, see the right red nerve path innervating the central and lateral maxillary incisors in Figure 1. Until now, treatment has included a preliminary bridge replacing the central incisor. In the near future, a final treatment must be decided upon replacing both right incisors.

### 2.2. Patient 2

**Figure 7:**
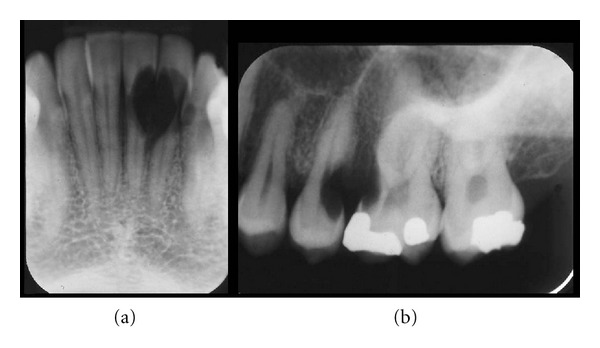
Patient 2: radiographs demonstrating regional aggressive root resorption in the left mandibular incisors (a) and in the left maxillary second premolar and first molar (b).

The patient, female born in 1974, was referred to the Department of Orthodontics in 2002 from a private dental practice for evaluation of resorption of the maxillary left second premolar and left first molar. Furthermore, severe resorption was observed in the mandibular left central and lateral incisors. The referring practitioner presumed that the resorption had occurred as a result of previous orthodontic treatment. The patient had previously received orthodontic treatment with removable appliance in the maxilla, but not with appliance in the lower jaw. Resorption due to orthodontic treatment was, therefore, discarded. The referring dentist requested elucidation of the aggressive resorption and a treatment plan (Figure 7). 

**Figure 8:**
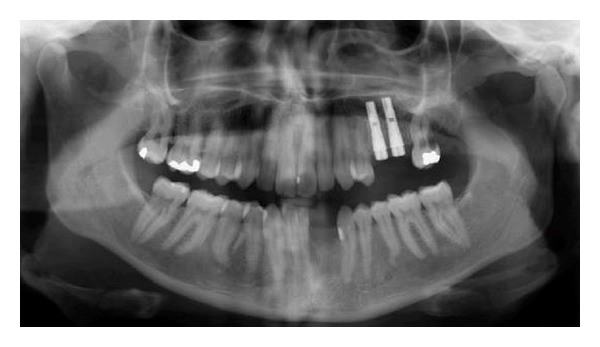
Patient 2: panoramic radiograph from 2004 demonstrating that the left mandibular incisors and left second maxillary premolar and first molar have been extracted. A filling is seen now in the mesial part of the cervical region at the left mandibular canine. Cervical resorption is observed distally in the left maxillary second molar. Implants have been inserted in the left maxillary premolar/molar region.

**Figure 9:**
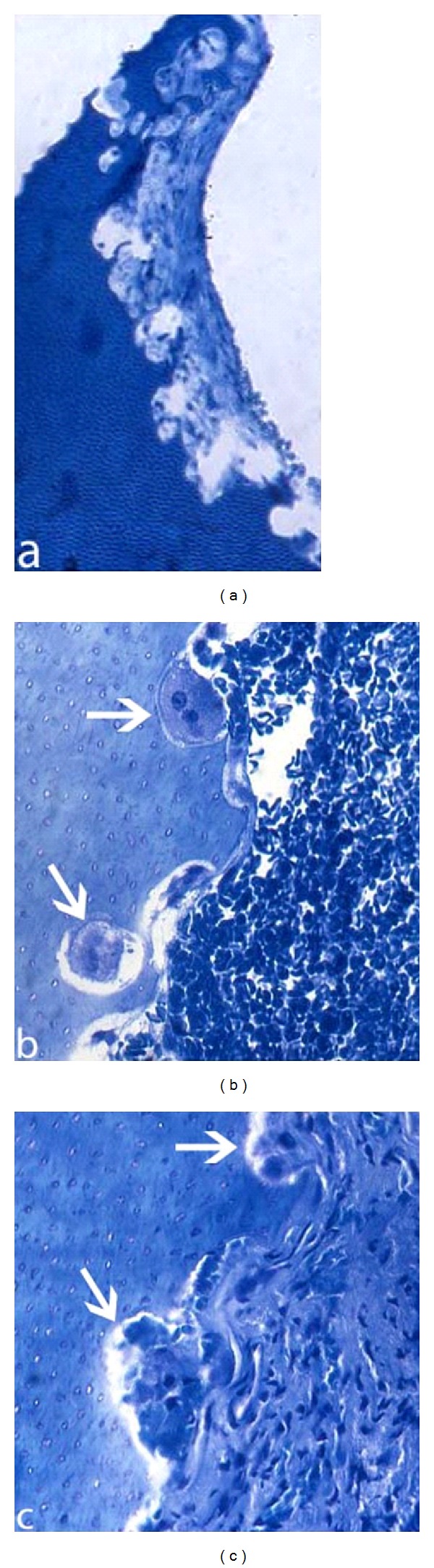
Patient 2: histological sections of different parts of the extracted mandibular left incisors, demonstrating aggressive multilacunary root surface (a). In a larger magnification, multinuclear resorptive dentinoclasts located in the lacunae are demonstrated and marked by arrows ((b) and (c)).

Different attempts to excavate and perform endodontic treatment and PA treatment of the two mandibular and two maxillary teeth failed, and in 2004 the maxillary premolar and molar as well as the incisors in the lower jaw were extracted (Figure 8). The extracted teeth were analyzed histologically. Large multinucleated resorptive cells situated in dentine lacunae were observed histologically (Figure 9). Panoramic radiograph from 2004 revealed the beginning of resorption distally in the maxillary left second molar, which was extracted shortly after the radiograph was taken. The left mandibular canine showed a slight cervical resorption, which was excavated and filled in 2004. 

The patient was examined twice a year since 2004 at the Copenhagen University Hospital. In 2006, the patient informed that she had been severely ill at the age of 9 and hospitalized for three months due to whooping cough and sequelae. No further information was available.

**Figure 10:**
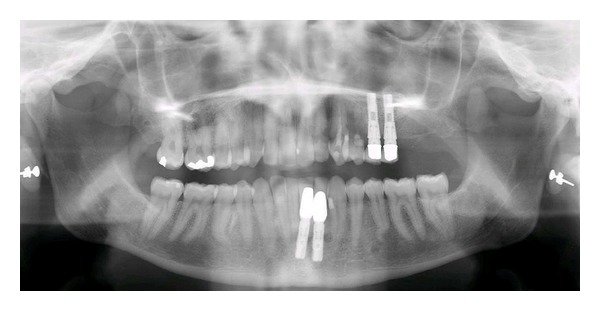
Patient 2: panoramic radiograph taken in 2007 demonstrating gradual resorption of the left-sided maxillary teeth when compared to the panoramic radiograph taken in 2004, demonstrated in Figure 8.

**Figure 11:**
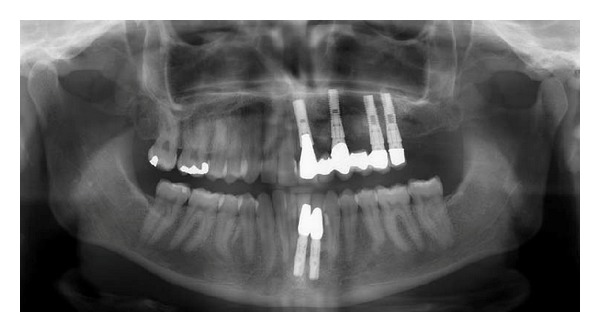
Patient 2: panoramic radiograph (from 2012) taken two years after the last implant had been inserted. The treatment has been performed by Chief Surgeon Nils Worsaae. The radiograph demonstrates that the destruction of teeth caused by virus attack on all left-sided maxillary peripheral nerves (illustrated in Figure 1) and on the left mandibular incisor nerve (marked red in Figure 1) has stopped. Also, the condition around the filling in the left mandibular canine is unchanged since 2004 (Figure 8).

**Figure 12:**
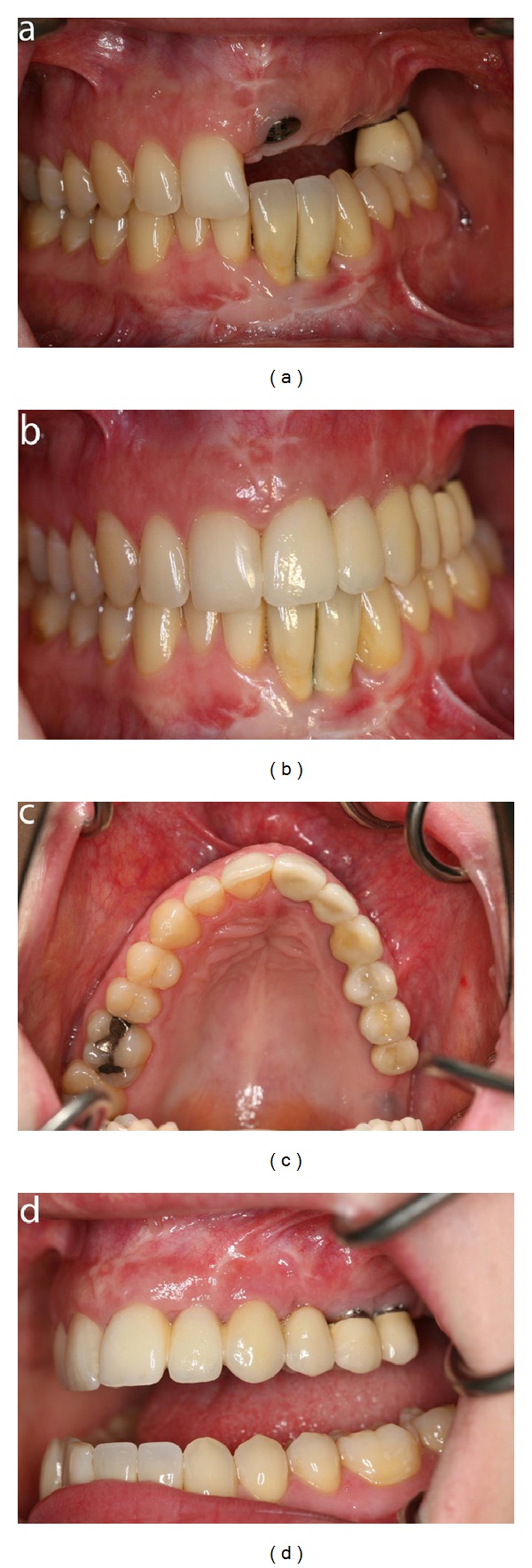
Patient 2: intraoral photographs demonstrating the dentition during and after treatment with dental implants. (a) Frontal view before crowns are inserted on the implants in the left maxillary incisor, canine, and first premolar region. Crowns have been inserted in the left mandibular incisor region. (b) Frontal view after insertion of crowns on the implants. (c) Occlusal view after insertion of crowns on the implants. (d) Lateral view demonstrating the crowns inserted in the implants in the left side of the maxilla.

During the following years all remaining maxillary permanent teeth in the left side showed cervical resorptions, which gradually became worse spreading along the entire root (Figure 10). This resulted in extractions. Since 2004, fixed implant supported dental reconstructions have been inserted in the left side of the maxilla, and in the incisor region in the mandible, two single implants have been inserted after bone grafting due to a narrow alveolar process (Figures 11 and 12). The condition around the filling in the left mandibular canine is unchanged since 2004 (Figure 11).

#### 2.2.1. Conclusion

The left-sided severe root resorptions in maxilla and mandible are presumably due to a neurogene spreading of the pertussis virus. According to the innervation pattern (Figure 1), no further destruction will occur in the maxilla. In the mandible, the slight resorption attack on the left canine will be followed, but as it has been unchanged in eight years, there is no reason to presume that further changes will occur here.

## 3. Discussion

Both cases show signs of neurogene spreading of virus initiating root resorption. In both cases, resorption began as invasive low cervical resorptions which later extended apically. Cervical root resorption has previously been described in association with periodontal disease [28]. 

The study does not demonstrate in which cell layer surrounding the root the resorption begins. The study does reveal that resorption occurs in regions determined by the innervation pattern and accordingly, that innervation seems to play a role in the resorption process. The study also reveals a correlation between the osseous development and root resorption. This is not surprising as it has been demonstrated that the osteoblastic function is regulated by the innervation [9].

The histological findings demonstrated in Patient 2 (Figure 10) are similar to that seen in Paget's disease [20]. However, the difference between Paget's disease and the cases presented in this study is that Paget's disease mostly affects the axial skeleton and has a strong genetic predisposition. In the present case, the root resorption is restricted to the innervation regions [27], and the anamnestic information in both cases suggests that the spreading of virus is the explanation. A regional resorption pattern related to the innervation pattern has not been described in Paget's disease.

It is perhaps the first time that such severe regional resorption processes are shown to be related to virus spreading along peripheral nerve paths. 

It is important in odontological diagnostics to distinguish between regional and general resorption because it is decisive for the treatment planning. The present cases demonstrate the importance of early diagnostics and careful registration of anamnestic information. For the orthodontist, it is particularly valuable to focus on idiopathic resorption, as demonstrated in case 2 where the cause behind resorption was wrongfully attributed to the orthodontic treatment. 

Registration of such cases may prove helpful for subsequent treatment advice when the number of patients registered has reached a sufficiently quantity.
